# Genetic analysis of pharmacogenomic VIP variants in the Wa population from Yunnan Province of China

**DOI:** 10.1186/s12863-021-00999-8

**Published:** 2021-11-19

**Authors:** Dandan Li, Linna Peng, Shishi Xing, Chunjuan He, Tianbo Jin

**Affiliations:** 1 Key Laboratory of Molecular Mechanism and Intervention Research for Plateau Diseases of Tibet Autonomous Region, School of Medicine, Xizang Minzu University, Xianyang, 712082 Shaanxi China; 2 Engineering Research Center of Tibetan Medicine Detection Technology, Ministry of Education, School of Medicine, Xizang Minzu University, Xianyang, 712082 Shaanxi China

**Keywords:** Pharmacogenomics, Wa, Genetic polymorphisms, VIP variants

## Abstract

**Background:**

The variation of drug responses and target does among individuals is mostly determined by genes. With the development of pharmacogenetics and pharmacogenomics, the differences in drug response between different races seem to be mainly caused by the genetic diversity of pharmacodynamics and pharmacokinetics genes. Very important pharmacogenetic (VIP) variants mean that genes or variants play important and vital roles in drug response, which have been listed in pharmacogenomics databases, such as Pharmacogenomics Knowledge Base (PharmGKB). The information of Chinese ethnic minorities such as the Wa ethnic group is scarce. This study aimed to uncover the significantly different loci in the Wa population in Yunnan Province of China from the perspective of pharmacogenomics, to provide a theoretical basis for the future medication guidance, and to ultimately achieve the best treatment in the future.

**Results:**

In this study, we recruited 200 unrelated healthy Wa adults from the Yunnan province of China, selected 52 VIP variants from the PharmGKB for genotyping. We also compared the genotype frequency and allele distribution of VIP variants between Wa population and the other 26 populations from the 1000 Genomes Project (http://www.1000Genomes.org/). Next, χ^2^ test was used to determine the significant points between these populations. The study results showed that compared with the other 26 population groups, five variants rs776746 (*CYP3A5*), rs4291 (*ACE*), rs3093105 *(CYP4F2*), rs1051298 (*SLC19A1*), and rs1065852 (*CYP2D6*) had higher frequencies in the Wa population. The genotype frequencies rs4291-TA, rs3093105-CA, rs1051298-AG and rs1065852-GA were higher than those of the other populations, and the allele distributions of rs4291-T and rs3093105-C were significantly different. Additionally, the difference between the Wa ethnic group and East Asian populations, such as CDX, CHB, and CHS, was the smallest.

**Conclusions:**

Our research results show that there is a significant difference in the distribution of VIP variants between the Wa ethnic group and the other 26 populations. The study results will have an effect on supplementing the pharmacogenomics information for the Wa population and providing a theoretical basis for individualised medication for the Wa population.

**Supplementary Information:**

The online version contains supplementary material available at 10.1186/s12863-021-00999-8.

## Background

Adverse drug reaction (ADR) having the ability of causing severe morbidity and mortality among patients is a major concern in clinical practice and the pharmaceutical industry. Increasing evidence shows that genetic differences between individuals are an important factor to ADR [1]. Pharmacogenomics is a discipline that studies how genetic factors affect the responses of individuals to drug therapy [2] and transforms the drug responses of individuals into a molecular diagnosis. Therefore, it can be used for individualised drug therapy [3]. Over the past 60 years, pharmacogenomics has been used to determine the genetic determinants of drug effects and to maximize drug efficacy and minimize ADR [1]. At present, it is necessary to integrate genomic data into the benefit and risk assessment of daily treatment so that individualised treatment has a certain possibility to vary from person to person [4].

PharmGKB, the Pharmacogenomics Knowledge Base (http://www.pharmgkb.org) is dedicated to disseminating information on how genetic variation causes variation in drug response. The PharmGKB database describes the connection between genes, diseases and drugs and provides various forms of knowledge, including the abstracts of very important pharmacogene (VIP) , drug pathway diagrams and selected literature notes [5]. The PharmGKB database also integrates information from the Clinical Pharmacogenetics Implementation Consortium (CPIC) to provide drug dosage guidance based on individual genotypes [6].

There are 56 ethnic groups recognized by the People's Republic of China, and different ethnic groups have different reactions to drugs. The Wa people reside mainly in the Yunnan Province of Southwestern China. The total population of the Wa ethnic group in China is 429,709, based on the data of the sixth nationwide population census in 2010. Because of the differences in genetics, physiology, pathology, diet, living environment, and nutritional status, the same drug regimen may not be suitable for every ethnic groups [7]. For example, in the Han, Bai, Wa, and Tibetan populations of the Yunnan Province in Southwestern China, there are significant differences in *MDR1* genotype distribution and the haplotype spectrum [8]. Studies have shown that *CYP2C9* mutation alleles frequencies in Caucasians are relatively higher (*2:12%, *3:8.3%), while *CYP2C9* mutation alleles frequencies in Chinese are relatively lower (CYP2C9*2:0%,*3:0%,*2:15%) [9]. Many of the observed drug response variability has a genetic basis, which is caused by the differences in the genetic determination of drug absorption, disposal, metabolism, or excretion [10].

We selected and genotyped 52 VIP variants among 27 genes in the Wa population. Next, we compared the genotype frequency and allelic distribution differences of VIP variants between the Wa ethnic group and the other 26 populations from the 1000 Genomes Project. The research results will expand the current Wa ethnic group pharmacogenomics information and ethnic diversity, and help clinicians to use genomic and molecular data to effectively implement personalized medicine in the future.

## Results

According to the PharmGKB database, we designed 67 SNPs and obtained 52 VIP variants, which are distributed mainly on 27 genes, mainly related to the cytochrome P450 family, dihydropyrimidine dehydrogenase, cyclooxygenase, N-acetyltransferase and others. The chromosome position, base pair, functional result, genotype-drug relationship, information about the drug related to gene mutation, gene, level of evidence, genotyping, minor allele frequency (MAF), and other basic information are shown in Table 1. The designed PCR primers is designed using the Agena MassARRAY Assay Design 4.0 software (San Diego, California, USA), and the specific information is showed in Supplementary Table 1.

**Table 1:** Basic characteristics of the selected VIP variants from the PharmGKB database and genotype frequencies in the Wa population

SNP ID	Chromosome	BP	Functional Consequence	Annotation	Molecules	Paper Discusses	Genes	Level of Evidence	Allele	MAF	Genotype		
											Mutation Homozygote	Heterozygote	Wild Homozygote
rs11572325	1	59896030	intron_variant				CYP2J2		T/A	0.043	0	17	183
rs10889160	1	59896449	intron_variant				CYP2J2		C/T	0.108	0	43	157
rs1760217	1	97137438	genic_downstream_transcript_variant,intron_variant	Genotypes AA + AG are associated with decreased survival when treated with antineoplastic agents in people with Pancreatic Neoplasms as compared to genotype GG.	antineoplastic agents	Efficacy	DPYD	3	G/A	0.330	24	84	92
rs1801159	1	97515839	coding_sequence_variant,genic_downstream_transcript_variant,intron_variant,missense_variant	Genotype TT is not associated with increased risk of Neutropenia when treated with cyclophosphamide, doxorubicin and fluorouracil in women with Breast Neoplasms as compared to genotypes CC + CT.	capecitabine/fluorouracil	Toxicity	DPYD	1A	C/T	0.265	10	85	103
rs1801265	1	97883329	non_coding_transcript_variant,intron_variant,coding_sequence_variant,5_prime_UTR_variant,missense_variant	Genotypes AA + AG is associated with decreased Drug Toxicity when treated with capecitabine or fluorouracil in people with Colorectal Neoplasms as compared to genotype GG.	fluorouracil/capecitabine	Toxicity	DPYD	1A	G/A	0.095	1	36	163
rs5275	1	186673926	3_prime_UTR_variant	Genotype AA is associated with increased progression-free survival and overall survival when treated with capecitabine and oxaliplatin in people with Colorectal Neoplasms as compared to genotypes AG + GG.	capecitabine oxaliplatin	Efficacy	PTGS2	3	G/A	0.175	3	64	133
rs20417	1	186681189	upstream_transcript_variant,non_coding_transcript_variant	Allele C is not associated with response to cetuximab or panitumumab in people with Colorectal Neoplasms as compared to allele G.	aspirin/ibuprofen/rofecoxib	Efficacy	PTGS2	3	G/C	0.003	0	1	199
rs12139527	1	201040054	missense_variant,coding_sequence_variant,intron_variant				CACNA1S		G/A	0.040	0	16	184
rs3850625	1	201047168	coding_sequence_variant,missense_variant				CACNA1S		A/G	0.003	0	1	199
rs2306238	1	237550803	intron_variant				RYR2		A/G	0.223	9	71	120
rs2231142	4	88131171	coding_sequence_variant,missense_variant	Genotypes GT + TT are not associated with increased likelihood of statin-related myopathy when treated with atorvastatin or simvastatin as compared to genotype GG.	rosuvastatin/rosuvastatin	Efficacy/Metabolism/PK	ABCG2	2A	T/G	0.196	7	64	128
rs2231137	4	88139962	coding_sequence_variant,missense_variant	Genotypes CT + TT is not associated with increased risk of Neutropenia when treated with valganciclovir in people with Kidney Transplantation as compared to genotype CC.	dasatinib imatinib nilotinib/irinotecan/imatinib	Other/Toxicity/Dosage	ABCG2	3	C/T	0.538	55	103	40
rs698	4	99339632	coding_sequence_variant,non_coding_transcript_variant,missense_variant	Genotype CT is associated with decreased likelihood of complete response when treated with cisplatin and cyclophosphamide in women with Ovarian Neoplasms as compared to genotypes CC + TT.	cisplatin cyclophosphamide	Efficacy	ADH1C	3	C/T	0.108	9	25	166
rs776746	7	99672916	intron_variant,splice_acceptor_variant,genic_downstream_transcript_variant,downstream_transcript_variant	Genotype CC is associated with decreased dose of tacrolimus in people with Kidney Transplantation as compared to genotypes CT + TT.	tacrolimus	Metabolism/PK	CYP3A5	1A	T/C	0.150	29	2	169
rs2242480	7	99763843	intron_variant	CYP3A4 *1G/*1G is associated with decreased metabolism of fentanyl in human liver microsomes as compared to CYP3A4 *1/*1 + *1/*1G.	tacrolimus	Metabolism/PK	CYP3A4	1B	T/C	0.337	18	98	83
rs1805123	7	150948446	missense_variant,coding_sequence_variant,genic_downstream_transcript_variant	Allele G is associated with decreased QT interval as compared to genotype TT.			KCNH2	3	G/T	0.095	0	38	162
rs4646244	8	18390208	upstream_transcript_variant,genic_upstream_transcript_variant,intron_variant	Allele A is associated with increased risk of Hepatitis when treated with ethambutol, isoniazid, pyrazinamide and rifampin in people with Tuberculosis.	ethambutol isoniazid pyrazinamide rifampin	Toxicity/Metabolism/PK	NAT2	3	A/T	0.223	13	62	122
rs4271002	8	18390758	upstream_transcript_variant,genic_upstream_transcript_variant,intron_variant	Allele C is associated with increased risk of intolerance of aspirin in people with Asthma as compared to allele G.	aspirin	Toxicity	NAT2	3	C/G	0.241	9	77	111
rs1041983	8	18400285	coding_sequence_variant,synonymous_variant	NAT2 *6A/*7B is associated with increased likelihood of Toxic liver disease when treated with isoniazid and rifampin in people with Tuberculosis.	ethambutol isoniazid pyrazinamide rifampin	Toxicity	NAT2	1B	T/C	0.455	42	96	60
rs1801280	8	18400344	missense_variant,coding_sequence_variant	NAT2 *5A is associated with increased risk of severe cutaneous adverse reactions when treated with sulfamethoxazole and trimethoprim in people with Acquired Immunodeficiency Syndrome.	ethambutol isoniazid pyrazinamide rifampin	Toxicity	NAT2	1B	C/T	0.043	0	17	183
rs1799929	8	18400484	coding_sequence_variant,synonymous_variant	Allele T is not associated with increased risk of hepatotoxicity when treated with ethambutol, isoniazid, pyrazinamide and rifampin in people with Tuberculosis as compared to allele C.	ethambutol isoniazid pyrazinamide rifampin	Toxicity	NAT2	1B	T/C	0.043	0	17	183
rs1799930	8	18400593	missense_variant,coding_sequence_variant	NAT2 *6/*7 is associated with increased likelihood of Toxic liver disease when treated with ethambutol, isoniazid, pyrazinamide and rifampin in people with Tuberculosis	ethambutol isoniazid pyrazinamide rifampin	Toxicity	NAT2	1B	A/G	0.234	12	69	118
rs1208	8	18400806	missense_variant,coding_sequence_variant	NAT2 *5B/*7B + *6A/*6A + *6A/*7B + *7B/*7B are associated with increased risk of Toxic liver disease when treated with ethambutol, isoniazid, pyrazinamide and rifampin in people with Tuberculosis.	ethambutol isoniazid pyrazinamide rifampin	Toxicity	NAT2	1B	G/A	0.043	0	17	183
rs1799931	8	18400860	missense_variant,coding_sequence_variant	NAT2 *6/*7 is associated with increased likelihood of Toxic liver disease when treated with ethambutol, isoniazid, pyrazinamide and rifampin in people with Tuberculosis.	ethambutol isoniazid pyrazinamide rifampin	Toxicity	NAT2	1B	A/G	0.230	10	72	118
rs1495741	8	18415371	None	Genotype AA is associated with increased likelihood of Toxic liver disease when treated with Drugs For Treatment Of Tuberculosis as compared to genotypes AG + GG.	Drugs For Treatment Of Tuberculosis	Toxicity	NAT2	3	G/A	0.370	26	93	77
rs2115819	10	45405641	intron_variant	Genotype GG is associated with increased FEV1 response when treated with montelukast in people with Asthma as compared to genotypes AA + AG.	montelukast	Efficacy	ALOX5	3	A/G	0.140	9	38	153
rs4244285	10	94781859	coding_sequence_variant,synonymous_variant	Allele A is associated with decreased exposure to clopidogrel active metabolite when treated with clopidogrel in healthy individuals as compared to allele G.	nelfinavir	Metabolism/PK	CYP2C19	3	A/G	0.389	31	92	75
rs1057910	10	94981296	missense_variant,coding_sequence_variant	CYP2C9 *1/*3 is associated with decreased metabolism of meloxicam in healthy individuals as compared to CYP2C9 *1/*1.	piroxicam	Metabolism/PK	CYP2C9	1A	C/A	0.023	0	9	191
rs11572103	10	95058349	missense_variant,coding_sequence_variant	CYP2C8 *1/*3 + *3/*3 is associated with increased response to paclitaxel in women with Breast Neoplasms as compared to CYP2C8 *1/*1.	rosiglitazone	Toxicity	CYP2C8	3	A/T	0.010	0	4	196
rs7909236	10	95069673	upstream_transcript_variant	Allele T is not associated with concentrations of imatinib in people with Neoplasms as compared to allele G.			CYP2C8		T/G	0.030	0	12	188
rs17110453	10	95069772	upstream_transcript_variant	Genotypes AC + CC is not associated with resistance to clopidogrel in people with Stroke as compared to genotype AA.			CYP2C8		C/A	0.363	26	93	81
rs3813867	10	133526101	non_coding_transcript_variant,upstream_transcript_variant	CYP2E1 *1/*5B is associated with increased elimination rate of acetaminophen in people with Liver Diseases, Alcoholic as compared to CYP2E1 *1/*1.	Drugs For Treatment Of Tuberculosis	Toxicity	CYP2E1	3	C/G	0.075	3	24	172
rs2031920	10	133526341	non_coding_transcript_variant,upstream_transcript_variant	Genotypes CT + TT are associated with increased risk of Toxic liver disease when treated with Drugs For Treatment Of Tuberculosis in people with Tuberculosis as compared to genotype CC.	Drugs For Treatment Of Tuberculosis		CYP2E1	3	T/C	0.098	3	33	164
rs6413432	10	133535040	intron_variant	Genotype TT is associated with increased progression-free survival when treated with cisplatin and cyclophosphamide in women with Ovarian Neoplasms as compared to genotype AT.	cisplatin cyclophosphamide	Efficacy	CYP2E1	3	A/T	0.025	0	10	190
rs2070676	10	133537633	intron_variant	Genotype CG is associated with increased risk of severe emesis when treated with cisplatin and cyclophosphamide in women with Ovarian Neoplasms as compared to genotype CC.	cisplatin cyclophosphamide	Efficacy/Toxicity	CYP2E1	3	G/C	0.175	3	64	133
rs5219	11	17388025	missense_variant,stop_gained,5_prime_UTR_variant,intron_variant,coding_sequence_variant	Allele T is associated with decreased activity of KCNJ11 when treated with glibenclamide pancreatic islet cells.	gliclazide	Efficacy	KCNJ11	3	T/C	0.340	9	118	73
rs1801028	11	113412762	missense_variant,coding_sequence_variant	Genotypes CG + GG are not associated with response to antipsychotics in people with Schizophrenia as compared to genotype CC.			DRD2		C/G	0.005	0	2	198
rs2306283	12	21176804	missense_variant,coding_sequence_variant	Genotype AA is associated with decreased response to rocuronium as compared to genotypes AG + GG.	pitavastatin	Metabolism/PK	SLCO1B1	3	A/G	0.168	5	57	138
rs4516035	12	47906043	upstream_transcript_variant	Allele T is associated with increased jejunal CYP3A4 protein levels as compared to allele C.	midazolam	Metabolism/PK	VDR	3	C/T	0.025	0	10	190
rs762551	15	74749576	intron_variant	CYP1A2 *1K is associated with decreased transcription of CYP1A2 when exposed to xenobiotics in B1642 cells.	caffeine	Toxicity	CYP1A2	3	C/A	0.321	17	91	87
rs2472304	15	74751897	intron_variant	Allele A is associated with increased likelihood of remission when treated with paroxetine in people with Depressive Disorder, Major as compared to allele G.	paroxetine/erlotinib	Efficacy/Metabolism/PK	CYP1A2	3	A/G	0.083	0	33	167
rs750155	16	28609251	5_prime_UTR_variant,intron_variant,genic_upstream_transcript_variant,upstream_transcript_variant	Allele T is not associated with ABT-751 pharmacokinetic parameters when treated with ABT-751 in people with Neoplasms as compared to allele C.			SULT1A1		T/C	0.406	22	112	58
rs1800764	17	63473168	None				ACE		C/T	0.269	14	79	106
rs4291	17	63476833	upstream_transcript_variant	Genotypes AT + TT are associated with increased risk of aspirin intolerance when exposed to aspirin in people with Asthma as compared to genotype AA.	captopril/aspirin/amlodipine chlorthalidone lisinopril	Efficacy/Toxicity/Efficacy	ACE	3	T/A	0.500	0	200	0
rs4267385	17	63506395	None	Genotypes CC + CT are associated with same protective properties against angiotensin-converting enzyme inhibitors-induced cough when treated with Ace Inhibitors, Plain in people with homozygous GG genotype for rs4343 as compared to genotype TT.	Ace Inhibitors Plain	Toxicity	ACE	3	T/C	0.260	16	71	111
rs2108622	19	15879621	missense_variant,coding_sequence_variant	CYP4F2 *1/*3 + *3/*3 are associated with increased exposure to Vitamin K1 in healthy individuals as compared to CYP4F2 *1/*1.	warfarin	Dosage	CYP4F2	1A	T/C	0.173	6	57	137
rs3093105	19	15897578	missense_variant,coding_sequence_variant	Allele C is associated with increased catalytic activity of CYP4F2 when treated with vitamin e in Sf9 insect cells transfected with CYP4F2 as compared to allele A.	vitamin e	Metabolism/PK	CYP4F2	3	C/A	0.497	1	198	0
rs8192726	19	40848591	intron_variant	Genotypes AC + CC are associated with increased plasma concentration (p=0.028) of efavirenz in people with HIV Infections as compared to genotype AA.	efavirenz	Other	CYP2A6	4	A/C	0.163	8	49	143
rs1051298	21	45514912	intron_variant,3_prime_UTR_variant	Allele G is associated with increased progression-free survival when treated with bevacizumab and pemetrexed in people with Lung Neoplasms as compared to allele A.	pemetrexed	Efficacy	SLC19A1	3	G/A	0.431	8	152	35
rs1051296	21	45514947	intron_variant,3_prime_UTR_variant				SLC19A1		A/C	0.469	44	95	56
rs1131596	21	45538002	missense_variant,5_prime_UTR_variant,synonymous_variant,genic_upstream_transcript_variant,coding_sequence_variant	Allele G is not associated with response to methotrexate in children with Precursor Cell Lymphoblastic Leukemia-Lymphoma as compared to allele A.			SLC19A1		G/A	0.490	33	129	37
rs1065852	22	42130692	intron_variant,missense_variant,coding_sequence_variant	Allele A is associated with decreased clearance of alpha-hydroxymetoprolol in healthy individuals as compared to allele G.	paroxetine	Metabolism/PK	CYP2D6	1A	G/A	0.430	29	170	1

*SNP* Single nucleotide polymorphism, *BP* Base pair, *MAF* Minor allele frequency

We used the chi-square test to study the frequency distribution of 52 loci and compared the Wa ethnic group with the other 26 different populations from the 1000 Genomes Project (CDX, CHB, CHS, JPT, KHV, ACB, ASW, ESN, GWD, LWK, MSL,YRI, CLM, MXL, PEL, PUR, CEU, FIN, GBR, IBS, TSI, BEB, GIH, ITU, PJL and STU). Compared with the other 26 ethnic groups, we observed 17, 21, 18, 22, 18, 33, 32, 36, 37, 33, 34, 36, 37, 33, 35, 38, 36, 40, 39, 41, 38, 32, 40, 39, 40, and 39 different SNPs without adjustment (*p* < 0.05) (Table 2). The table shows that the Wa ethnic group has the smallest difference compared with the CDX, CHB, CHS, and KHV in the East Asian population, but the biggest difference is in the GIH and PJL in the South Asian population compared with the FIN and IBS in the European population. Among these loci, *CYP3A5* rs776746, *ACE* rs4291, *CYP4F2* rs3093105, *SLC19A1* rs1051298, and *CYP2D6* rs1065852 had higher frequencies compared with the other 26 populations. We also found that the significant differences between KHV, JPT, CDX, LWK and Wa people were in rs3093105 and rs1065852.

**Table 2:** Significant VIP variants in the Wa people compared with the other 26 populations without adjustment

SNP ID	Genes	p<0.05			EAS		AFR							AMR				EUR					SAS				
		CDX	CHB	CHS	JPT	KHV	ACB	ASW	ESN	GWD	LWK	MSL	YRI	CLM	MXL	PEL	PUR	CEU	FIN	GBR	IBS	TSI	BEB	GIH	ITU	PJL	STU
rs11572325	*CYP2J2*		0.306375642				1.02E-06*	5.45E-06*	1.90E-06*	4.42E-06*	2.08E-05*	8.09E-05*	2.75E-06*	0.005284096*			7.76E-06*	0.009360496*	6.62E-05*	0.066258015	0.023683277*	0.011905569*			0.298107259		
rs10889160	*CYP2J2*	0.329164673	0.002444771*	0.028881237*	0.000294247*	0.040383079*	5.88E-19*	1.08E-10*	1.52E-21*	8.72E-15*	1.62E-16*	9.67E-19*	4.46E-21*	0.113681618			0.000148487*	0.130780521	9.14E-06*	0.248614483	0.006404332*	0.108048571			0.028992767*	0.271827376	
rs1760217	*DPYD*	0.591174047	0.635254374	0.043390478*	0.285254147	0.098440081	0.000830268*	0.083572784	0.020342952*	1.07E-05*	0.001081997*	0.013652794*	5.51E-05*	**0.00026507**	0.424945255	0.003384269*	3.19E-06*	0.056397577	2.18E-05*	5.35E-05*	**0.00022263**	0.00048027*	0.002729039*	0.021913163*	9.86E-09*	1.10E-05*	3.54E-08*
rs1801159	*DPYD*	0.88117013	0.950798386	0.002842988*	0.238643736	0.223227528	0.001091502*	0.018913811*	0.028486512*	6.33E-08*	0.520772569	3.62E-07*	0.002338955*	0.090969695	0.648090677	2.14E-06*	0.118531739	0.007956605*	0.000828883*	0.022882805*	0.160795921	0.068964677	1.76E-05*	8.42E-06*	4.31E-10*	3.16E-06*	2.57E-08*
rs1801265	*DPYD*	0.052749931	0.469459751	0.537814287	0.14153267	0.75955499	1.55E-14*	9.57E-19*	7.19E-19*	7.68E-22*	2.18E-22*	1.80E-14*	5.20E-20*	1.97E-05*	3.98E-05*	0.036349573*	8.12E-07*	0.0582413	2.03E-09*	0.010507456*	0.000166496*	0.000111818*	0.004339857*	5.32E-12*	3.10E-10*	5.62E-09*	0.001731231*
rs5275	*PTGS2*	0.336758749	0.417665708	0.961588522	0.118298167	0.470903061	5.37E-24*	3.85E-17*	2.15E-28*	4.85E-23*	3.89E-24*	6.60E-27*	1.08E-28*	2.24E-08*	7.96E-06*	4.18E-08*	7.32E-05*	9.47E-08*	0.016894923*	0.000783464*	2.20E-05*	0.000269579*	3.18E-08*	8.54E-08*	1.31E-06*	2.86E-13*	7.33E-09*
rs20417	*PTGS2*						**5.38E-29**	**1.81E-26**	**1.16E-37**	**3.26E-27**	**2.82E-24**	**3.93E-36**	**5.95E-33**	**2.31E-19**	**3.44E-19**	**6.57E-17**	**1.57E-18**	**5.14E-15**	**2.82E-09**	**2.64E-12**	**2.26E-13**	**2.04E-16**	**1.85E-15**	**1.81E-15**	**1.45E-14**	**1.54E-19**	**8.50E-18**
rs12139527	*CACNA1S*	0.010930581*	0.005747244*	0.003350606*	0.001024703*		9.11E-34*	1.29E-29*	1.83E-41*	2.75E-43*	5.74E-36*	7.83E-41*	3.31E-41*				4.94E-08*	0.001983601*	0.003138048*	0.015381705*	0.002233347*	0.038502751*		0.229113935		0.214498852	0.205806221
rs3850625	*CACNA1S*													9.08E-10*		4.71E-09*		2.62E-07*	4.14E-16*	2.62E-12*	1.13E-07*	4.73E-09*	5.03E-13*	4.51E-25*	2.99E-17*	4.65E-16*	1.61E-13*
rs2306238	*RYR2*	0.243008296	0.511988338	0.32067139	0.291217242	0.427860312	2.93E-06*	0.001321721*	7.38E-09*	1.07E-07*	5.88E-07*	5.76E-08*	3.11E-07*	0.315347468	0.004033895*	0.000709533*	0.049208479*	0.594776616	0.767314427	0.934677486	0.948399042	0.944870157	0.468046737	0.533568812	0.00731298*	0.031525792*	0.012025534*
rs2231142	*ABCG2*	0.759304079	0.006272733*	0.18134526	0.001634302*	0.00036571*	2.96E-09*	0.006292525*	8.53E-11*	3.58E-10*	8.53E-11*	1.12E-07*	1.30E-11*	0.093444383	0.910555607	0.363795593	0.016893966*	0.04814984*	0.003955761*	0.197481137	0.000186352*	2.64E-05*	0.098393345	0.0001759*	0.016749365*	0.015307606*	0.00252365*
rs2231137	*ABCG2*	0.03195508*	2.53E-07*	1.10E-05*	4.49E-17*	0.0021976*	3.93E-29*	6.69E-25*	2.40E-29*	2.26E-33*	1.72E-21*	2.40E-22*	4.17E-30*	4.32E-18*	3.98E-05*	0.21099732	8.12E-07*	1.43E-31*	2.87E-24*	2.14E-30*	1.47E-30*	7.16E-27*	1.36E-10*	7.44E-19*	9.18E-24*	1.79E-22*	1.55E-14*
rs698	*ADH1C*	0.014000323*	0.063405752	0.252382783	0.258458735	0.077767236	0.313321517	0.224283945	0.281282137	0.039819211*	0.004885396*	0.063702796	0.080263228	6.40E-05*	7.96E-06*	0.001138652*	7.32E-05*	1.65E-21*	1.19E-20*	1.04E-15*	1.26E-08*	1.53E-09*	0.000640869*	2.30E-07*	4.56E-06*	8.44E-11*	6.86E-12*
rs776746	*CYP3A5*	1.44E-20*	1.5E-18*	1.25E-18*	1.82E-21*	5.24E-21*	1.18E-37*	1.94E-33*	3.90E-43*	2.02E-40*	2.85E-40*	8.23E-39*	4.59E-45*	2.21E-13*	3.44E-19*	1.43E-09*	1.57E-18*	4.33E-06*	2.47E-06*	6.55E-07*	8.03E-08*	3.20E-07*	3.69E-22*	3.61E-20*	5.26E-21*	1.44E-19*	1.24E-20*
rs2242480	*CYP3A4*	0.174181201	0.021891099*	0.023739907*	0.23406711	0.435345164	2.62E-23*	1.64E-15*	3.89E-36*	1.69E-28*	1.47E-36*	6.57E-33*	1.50E-30*	0.409092294	0.192100104	1.35E-07*	0.08254084	6.01E-14*	3.60E-12*	5.94E-12*	2.02E-09*	1.22E-11*	0.17415559	0.803974761	0.115551578	0.089908531	0.456718839
rs1805123	*KCNH2*		0.001573873*											0.000823584*	0.056854534		1.21E-05*	1.31E-06*	0.004686145*	1.77E-07*	3.87E-08*	2.29E-09*	2.02E-09*	1.52E-06*	2.17E-08*	0.00023004*	2.36E-05*
rs4646244	*NAT2*	0.908457449	0.610136371	0.315761804	0.54419548	0.005511981*	0.539375881	0.681282207	0.569019305	0.731729318	0.470793074	0.766246664	0.573758645	0.765141605	0.045785995*	0.001228669*	0.743776944	0.059300297	0.228557873	0.287926694	0.380483065	0.197043557	0.719514347	0.000339744*	0.016243392*	**0.0063584**	3.97E-05*
rs4271002	*NAT2*	0.066715616	0.189042432	0.327040513	0.208044975	0.299878957	0.000159278*	0.007107265*	3.30E-10*	3.72E-10*	2.07E-05*	0.000330686*	4.77E-06*	0.000635858*	0.417562363	0.072199748	0.357070744	1.74E-06*	0.001117691*	0.077554184	0.006521172*	0.060691113	0.476861068	0.006951173*	0.000332936*	0.008015007*	0.008719915*
rs1041983	*NAT2*	0.3	0.101689882	0.998513527	0.036626279*	0.132118736	0.629462005	0.990184178	0.158209247	0.24464648	0.844220999	0.183124559	0.610133391	0.007677766*	0.001717351*	1.66E-05*	0.001063258*	0.00091851*	0.000817232*	0.001042721*	0.010149174*	0.001176575*	0.155505108	0.951353573	0.383411457	0.677678934	0.579411379
rs1801280	*NAT2*					0.356035128	4.89E-14*	4.38E-14*	**2.97E-14**	**6.83E-19**	**9.29E-22**	**4.03E-11**	**2.25E-11**	**2.47E-21**	**8.41E-19**	**6.89E-15**	**4.17E-22**	**2.27E-25**	**1.72E-27**	**3.07E-27**	**1.97E-30**	**1.73E-26**	**4.00E-19**	**1.06E-18**	**8.10E-20**	**6.31E-25**	**3.64E-15**
rs1799929	*NAT2*					0.361199143	4.69E-11*	5.21E-11*	1.75E-08*	1.49E-15*	8.89E-19*	9.21E-08*	2.85E-06*	4.54E-20*	4.43E-18*	3.32E-14*	2.50E-19*	3.12E-25*	5.32E-26*	9.84E-26*	2.87E-30*	1.73E-26*	6.28E-18*	3.27E-16*	5.98E-18*	9.49E-22*	3.69E-14*
rs1799930	*NAT2*	0.972002065	0.618005239	0.751795155	0.694537677	0.012320182*	0.69375469	0.192800923	0.730809	0.372603522	0.412257221	0.783101212	0.584057039	0.881554266	0.05609109	0.000190302*	0.758530406	0.111309329	0.239563334	0.199562595	0.259685706	0.456150126	0.660367076	0.000563153*	0.020071798*	0.004077679*	1.56E-05*
rs1208	*NAT2*					0.323358729	1.18E-20*	2.46E-17*	3.46E-22*	1.32E-27*	1.45E-27*	4E-19*	2.65E-22*	6.25E-22*	4.16E-23*	6.89E-15*	5.00E-22*	6.43E-24*	1.92E-25*	8.03E-26*	1.73E-30*	4.24E-27*	9.85E-22*	1.06E-18*	9.54E-20*	1.22E-25*	2.87E-17*
rs1799931	*NAT2*	0.184796686	0.049117201*	0.317619594	0.00015301*	0.47411742	2.09E-08*	3.35E-05*	6.27E-11*	8.62E-12*	1.51E-11*	8.44E-07*	4.04E-09*	7.83E-06*	0.068024303	0.013692827*	2.98E-05*	3.40E-12*	1.03E-08*	1.72E-09*	1.60E-09*	3.59E-11*	0.00114834*	2.94E-07*	9.44E-07*	1.38E-06*	1.99E-05*
rs1495741	*NAT2*	0.050954298	3.07932E-07*	0.011465852*	5.51E-08*	0.65417351	0.507903694	0.486579857	0.257460454	0.281747884	0.696434574	0.119487491	0.019989993*	0.041821768*	0.67785097	0.070175871	0.248664031	0.043637899*	0.00729876*	0.003075161*	5.75E-05*	0.029405924*	0.055676203	0.000771028*	0.011226574*	6.30E-06*	7.45E-05*
rs2115819	*ALOX5*	0.002595851*	0.000362646*	0.071696502	0.008651836*	0.093741999	1.65E-37*	8.62E-25*	4.45E-37*	5.67E-41*	9.79E-33*	1.05E-31*	5.94E-40*	3.77E-16*	9.55E-11*	9.96E-07*	1.12E-14*	5.62E-24*	3.92E-19*	3.07E-18*	6.98E-20*	8.90E-21*	9.03E-16*	6.62E-23*	1.72E-20*	9.48E-16*	2.24E-14*
rs4244285	*CYP2C19*	0.011385106*	0.084348706	0.104324626	0.277350778	0.037979119*	8.78E-08*	2.52E-06*	6.75E-05*	3.23E-10*	0.000120419*	6.92E-06*	1.11E-07*	9.77E-11*	2.52E-07*	4.15E-14*	8.47E-10*	2.17E-09*	0.000116625*	5.20E-08*	7.41E-09*	4.51E-13*	0.357083431	0.187693423	0.922883969	0.176804078	0.86504652
rs1057910	*CYP2C9*			0.199109143																		0.00223127		5.21E-07*	0.000111245*	0.000272478*	
rs11572103	*CYP2C8*						1.57E-16*	5.93E-10*	7.08E-15*	8.71E-19*	3.56E-09*	5.00E-12*	1.60E-14*														
rs7909236	*CYP2C8*		0.001111119			0.284095031								6.35E-18*	1.17E-16*	1.77E-22*	1.70E-08*	1.45E-16*	3.59E-15*	1.16E-11*	3.98E-10*	2.96E-09*	1.61E-09*	3.50E-14*	3.03E-12*	1.49E-09*	3.17E-06*
rs17110453	*CYP2C8*	0.021440387*	0.826534465	0.858474033	0.794091714	0.043147139*	6.00E-20*	2.07E-14*	1.87E-20*	2.78E-24*	5.66E-22*	1.40E-19*	1.07E-22*	8.29E-11*	2.41E-07*	5.14E-14*	5.74E-10*	1.31E-11*	0.000358179*	6.93E-10*	0.000120667*	4.47E-11*	0.391440466	0.295282612	0.706742958	0.058493	0.76290557
rs3813867	*CYP2E1*	0.006552652*	3.59E-06*	0.000102114*	0.000102412*	**2.78E-06**	0.742811481	0.550417961	0.305302386	0.279166336	0.068293812	0.914073362	0.730517163	0.044163386*	0.009317049*	0.003053677*	0.452117467	0.47034354	0.246502725	0.104281398	0.044268805*	0.388354635	0.01515297*	0.004894979*	0.00523142*	0.007795656*	0.001761454*
rs2031920	*CYP2E1*	0.093771053	8.07E-05*	0.003359719*	0.004926448*	0.000300332*	5.35E-05*	0.016624442*	3.99E-05*	1.02E-05*	3.99E-05*	0.00015751*	1.66E-05*	0.253219685	0.120657802	0.09925265	0.219031776	0.272358474	0.037336905*	0.015728559*	0.004271931*	0.136905408	0.001606451*	0.000331478*	0.000363614*	0.000634024*	0.000111032*
rs6413432	*CYP2E1*	4.2E-19*	1.31E-16*	1.45E-15*	3.14E-16*	1.66E-19*			0.003915588*	0.000270607*		0.065854286	0.000530663*	6.57E-10*	4.59E-08*	9.02E-09*	2.97E-09*	1.63E-06*	1.26E-05*	0.015544772*	0.008287334*	9.51E-05*	8.46E-12*	1.83E-17*	7.76E-12*	9.13E-08*	2.51E-12*
rs2070676	*CYP2E1*	0.002553305*	0.827109248	0.672645828	0.614168132	0.448640164	3.81E-30*	1.06E-17*	1.00E-28*	5.52E-30*	2.70E-35*	3.78E-31*	7.09E-28*	0.290749722	0.022443644*	0.031891018*	0.002072634*	0.359414667	0.006774321*	0.034512086*	0.365848056	0.435369755	0.859781523	0.467538122	0.648934849	0.840697344	0.330668697
rs5219	*KCNJ11*	0.003978161*	0.000521662*	0.073272148	0.026139453*	0.041995705*	4.08E-16*	5.26E-07*	1.86E-24*	2.18E-25*	1.16E-23*	7.22E-22*	4.56E-26*	6.22E-05*	0.030526542*	0.289021265	0.002118976*	0.171703986	0.003250285*	0.047623834*	0.008845052*	0.005988539*	0.000383538*	0.000294121*	0.00355488*	3.67E-07*	0.023357634*
rs1801028	*DRD2*		0.002743098*	0.003159452*														0.032791444*						1.31E-13*			9.84E-09*
rs2306283	*SLCO1B1*	0.658512672	0.205950281	0.516381109	9.67E-06*	0.255712933	0.343484593	0.092030119	0.155670314	0.421337787	0.240886061	0.739594068	0.842983386	8.87E-16*	5.47E-20*	2.85E-16*	5.37E-13*	1.50E-21*	3.18E-19*	3.33E-24*	2.97E-22*	4.46E-25*	3.98E-10*	1.37E-11*	7.78E-09*	1.72E-17*	2.41E-11*
rs4516035	*VDR*							0.000250973*						1.88E-18*	9.22E-15*	3.10E-09*	7.20E-25*	2.88E-25*	7.28E-39*	1.17E-28*	3.93E-26*	4.93E-31*	1.74E-10*	3.70E-11*	1.81E-10*	1.72E-15*	7.16E-13*
rs762551	*CYP1A2*	0.1	0.300829362	0.634709813	0.103718477	0.314988006	0.124745263	0.69186992	0.000364292*	0.028368256*	1.00E-05*	0.037262669*	0.002040475*	0.209128456	0.032953675*	1.13E-05*	0.76149116	0.340413414	0.468062248	0.230946324	0.358933662	0.230526214	0.035152873*	0.000510748*	0.001980774*	0.003245356*	2.42E-05*
rs2472304	*CYP1A2*	0.014236939*	0.257348035	0.001901901*	1.73E-05*	0.004237979*	0.103421545							2.83E-16*	1.62E-07*	0.189014451	2.04E-24*	2.27E-36*	7.10E-29*	1.44E-34*	2.50E-33*	1.03E-26*	0.000626231*	0.011964466*	0.071150838	2.56E-06*	0.290829511
rs750155	*SULT1A1*	0.056990114	0.141351868	0.016733876*	0.351244062	0.298586001	0.550366807	0.321948571	0.002066135*	0.000560201*	0.465867078	0.097094955	0.096184189	0.005138686*	0.028868337*	3.18E-23*	0.026490062*	0.371180365	0.071724101	0.22160908	0.807754642	0.357468926	0.000453366*	0.003608286*	0.003408433*	0.049227902*	1.96E-07*
rs1800764	*ACE*	0.056726465	0.39143495	0.217109705	5.34E-05*	0.045931922*	2.61E-33*	8.82E-23*	1.09E-40*	1.94E-46*	1.05E-35*	2.63E-43*	7.78E-46*	4.25E-05*	0.389243603	0.47000884	1.34E-05*	6.39E-06*	4.04E-05*	0.000366475*	0.000414932*	8.38E-08*	0.016211851*	3.48E-05*	0.002243978*	0.011118101*	0.021050251*
rs4291	*ACE*	3.00E-30*	1.28E-35*	5.55E-31*	2.57E-26*	4.86E-35*	1.33E-28*	1.67E-29*	2.72E-30*	8.30E-26*	4.86E-35*	6.79E-26*	3.69E-35*	3.10E-29*	1.28E-34*	4.75E-42*	9.11E-29*	2.72E-30*	1.42E-24*	1.18E-31*	6.37E-28*	5.39E-31*	3.96E-29*	8.23E-28*	2.65E-35*	6.22E-30*	1.00E-28*
rs4267385	*ACE*	0.60706182	0.896137823	0.304531817	0.437096269	0.448897001	1.22E-24*	2.40E-15*	3.40E-26*	4.89E-32*	2.33E-33*	3.63E-30*	4.04E-30*	5.61E-06*	0.001132547*	0.876941984	3.09E-09*	2.69E-10*	1.64E-09*	1.40E-11*	1.79E-12*	7.04E-21*	0.070967336	0.001307242*	0.196898102	0.000954545*	0.008151391*
rs2108622	*CYP4F2*	0.688253222	0.353058471	0.579831407	0.049533921*	0.229799176	0.085598381	0.082954611	4.34E-05*	0.000977448*	0.144761136	0.129717659	0.000161974*	0.010322998*	0.071572406	0.162826967	0.004590455*	0.075664532	0.39622905	0.005888374*	2.04E-06*	2.69E-05*	1.82E-08*	1.99E-10*	2.68E-08*	1.38E-07*	1.96E-09*
rs3093105	*CYP4F2*	1.60E-55*	3.54E-51*	3.51E-54*	1.79E-58*	8.09E-59*	1.14E-36*	1.95E-29*	4.42E-32*	2.15E-40*	4.26E-37*	2.84E-35*	3.52E-34*	2.57E-43*	1.25E-41*	7.05E-55*	1.53E-43*	7.84E-44*	8.51E-49	1.60E-40*	6.40E-34*	1.29E-38*	4.65E-44*	1.27E-45*	1.55E-41*	1.48E-41*	1.25E-45*
rs8192726	*CYP2A6*	0.71094678	0.565911012	0.311301082	0.390661417	0.305375625	0.004591427*	0.245752554	0.104409227	0.002175155*	0.073757598	0.010662896*	0.10444763	0.000189151*	0.006056478*	0.00149952*	0.000247622*	0.001004263*	0.129515608	0.000373767*	0.000723061*	0.004379184*	0.496057104	0.602716704	0.243968045	0.892476883	0.243968045
rs1051298	*SLC19A1*	1.51E-07*	2.66E-09*	0.000482386*	3.97E-08*	2.44897E-06*	7.14E-08*	1.60E-06*	2.36E-06*	3.37E-11*	4.68E-09*	4.73E-07*	4.55E-07*	6.37E-09*	1.09E-15*	8.09E-15*	6.04E-09*	6.48E-11*	5.13E-09	5.50E-14*	4.51E-11*	2.71E-10*	1.94E-06*	7.95E-08*	2.61E-07*	3.27E-07*	2.79E-09*
rs1051296	*SLC19A1*	0.609205002	0.771425657	0.124768121	0.752914304	0.254101023	0.503862027	0.471517174	0.162301179	0.644086821	0.581333718	0.868173465	0.468804993	0.434021469	**0.00179709**	**0.0062769**	0.216005729	**0.01134575**	0.243843261	**0.01625569**	0.616464101	0.080496685	0.640656314	0.903856176	0.257373002	0.581655047	0.830821988
rs1131596	*SLC19A1*	0.022919018*	0.023544228*	0.400775632	0.005294873*	0.131694616	6.91E-11*	0.004236642*	0.000115013*	2.60E-16*	1.59E-12*	7.50E-11*	4.12E-10*	0.021820836*	2.88E-06*	8.07E-05*	0.01622849*	0.161041225	0.045935608*	9.78E-06*	0.000809244*	0.006656253*	0.01182718*	0.003770171*	0.002710073*	0.000670055*	0.002377152*
rs1065852	*CYP2D6*	**4.20E-16**	1.82E-13*	1.88E-17*	6.28E-22*	7.67E-12*	4.99E-42*	2.82E-38*	3.62E-50*	2.33E-48*	4.31E-58*	2.22E-39*	1.39E-48*	2.14E-38*	1.76E-37*	8.68E-51*	8.43E-41*	4.66E-30*	3.71E-42*	1.47E-31*	2.16E-39*	2.84E-35*	1.95E-31*	3.30E-41*	1.17E-38*	6.16E-47*	2.88E-42*

*EAS* East Asian, *AFR* African, *AMR* American, *EUR* European, *SAS* South Asian, *ACB* African Caribbean in Barbados, *ASW* African Ancestry in Southwest US, *ESN* Esan in Nigeria, *GWD* Gambian in Western Divisions, The Gambia – Madinka, *LWK* Luhya in Webuye, Kenya, *MSL* Mende in Sierra Leone, *CLM* Colombian in Medellin, Colombia, *MXL* Mexican Ancestry in Los Angeles, California, *PEL* Peruvian in Lima, Peru, *PUR* Puerto Rican in Puerto Rico, *CDX* Chinese Dai in Xishuangbanna, China, *YRI* Yoruba in Ibadan, Nigeria, *CHS* Han Chinese South, *JPT* Japanese in Tokyo, Japan, *KHV* Kinh in Ho Chi Minh City, Vietnam, *CEU* Utah residents with Northern and Western European ancestry, *FIN* Finnish in Finland, *GBR* British in England and Scotland, *IBS* Iberian populations in Spain, *TSI* Toscani in Italy, *BEB* Bengali in Bangladesh, *GIH* Gujarati Indians in Houston, Texas, *ITU* Indian Telugu in the UK, *PJL* Punjabi in Lahore, Pakistan, *STU* Sri Lankan Tamil in the UK, *CHB* Han Chinese in Beijing, China

*p* values were calculated from χ^2^ test

Bold indicates*p < 0.05 indicates statistical significance

Compared the Wa ethnic group with the other 26 population groups, there were 6, 9, 6, 10, 7, 28, 25, 27, 32, 29, 28, 30, 23, 21, 23, 27, 27, 24, 24, 24, 26, 20, 26, 24, 26, and 27 different VIP variants after Bonferroni's multiple adjustments (*p* < 0.05/(52×26)) (Table 3). Compared with the Wa population in the Yunnan province of China, the differences of CDX, CHB, and CHS the East Asian population are the smallest; the differences of GWD, LWK, and YRI, whose genomes are African, are the biggest. *CYP3A5* rs776746, *ACE* rs4291, *CYP4F2* rs3093105, *SLC19A1* rs1051298, and *CYP2D6* rs1065852 in the Wa population still have a high frequency in the other 26 populations after adjustment. There are also some variants becoming insignificant, such as *NAT2* rs4646244 and *CYP2A6* rs8192726. According to statistics, the frequency of *NAT2* rs1041983, rs1799930 and *CYP2C9* rs1057910 among the Wa population is only different from PEL, STU, and GIH, while other loci are different between the Wa and multiple ethnic groups.

**Table 3:** Significant VIP variants in the Wa people compared with the other 26 populations after Bonferroni’s multiple adjustment

SNP ID	Genes	*p* < 0.05/(52×26)				EAS	AFR							AMR				EUR					SAS				
		CDX	CHB	CHS	JPT	KHV	ACB	ASW	ESN	GWD	LWK	MSL	YRI	CLM	MXL	PEL	PUR	CEU	FIN	GBR	IBS	TSI	BEB	GIH	ITU	PJL	STU
rs11572325	CYP2J2						1.02E-06*	5.45E-06*	1.90E-06*	4.42E-06*	2.08E-05*	8.09E-05	**2.75E-0**6*				7.76E-06*		6.62E-05								
rs10889160	CYP2J2				0.000294		5.88E-19*	1.08E-10*	1.52E-21*	8.72E-15*	1.62E-16*	9.67E-19*	4.46E-21*				0.000148		9.14E-06*								
rs1760217	DPYD						0.00083			1.07E-05*			5.51E-05	0.000265			3.19E-06*		2.18E-05*	5.35E-05	0.000223	0.00048			9.86E-09*	1.1E-05*	3.54E-08*
rs1801159	DPYD									6.33E-08*		3.62E-07*				2.14E-06*			0.000829				1.76E-05*	8.42E-06*	4.32E-10*	3.16E-06*	2.57E-08*
rs1801265	DPYD						1.55E-14*	9.57E-19*	7.19E-19*	7.68E-22*	2.18E-22*	1.8E-14*	5.2E-20*	1.97E-05*	3.98E-05		8.12E-07*		2.03E-09*		0.000166	0.000112		5.32E-12*	3.1E-10*	5.62E-09*	
rs5275	PTGS2						5.37E-24*	3.85E-17*	2.15E-28*	4.85E-23*	3.89E-24*	6.6E-27*	1.08E-28*	2.24E-08*	7.96E-06*	4.18E-08*	7.32E-05	9.47E-08*		0.000783	2.2E-05*	0.00027	3.18E-08*	8.54E-08*	1.31E-06*	2.86E-13*	7.33E-09*
rs20417	PTGS2						5.38E-29*	1.81E-26*	1.16E-37*	3.26E-27*	2.82E-24*	3.93E-36*	5.95E-33*	2.31E-19*	3.44E-19*	6.57E-17*	1.57E-18*	5.14E-15*	2.82E-09*	2.64E-12*	2.26E-13*	2.04E-16*	1.85E-15*	1.81E-15*	1.45E-14*	1.54E-19*	8.50E-18*
rs12139527	CACNA1S						9.11E-34*	1.29E-29*	1.83E-41*	2.75E-43*	5.74E-36*	7.83E-41*	3.31E-41*				4.94E-08										
rs3850625	CACNA1S													9.08E-10*		4.71E-09*		2.62E-07*	4.14E-16*	2.62E-12*	1.13E-07*	4.73E-09*	5.03E-13*	4.51E-25*	2.99E-17*	4.65E-16*	1.61E-13*
rs2306238	RYR2						2.93E-06*		7.38E-09*	1.07E-07*	5.88E-07*	5.76E-08*	3.11E-07*			0.00071											
rs2231142	ABCG2					0.000366	2.96E-09*		8.53E-11*	3.58E-10*	8.53E-11*	1.12E-07*	1.3E-11*								0.000186	2.64E-05*		0.000176			
**rs2231137**	ABCG2		2.53E-07*	1.1E-05*	4.49E-17*		3.93E-29*	6.69E-25*	2.4E-29*	2.26E-33*	1.72E-21*	2.4E-22*	4.17E-30*	4.32E-18*	2.82E-10*		1.26E-18*	1.43E-31*	2.87E-24*	2.14E-30*	1.47E-30*	7.16E-27*	1.36E-10*	7.44E-19*	9.18E-24*	1.79E-22*	1.55E-14*
rs698	ADH1C													6.41E-05	1.92E-07*		2.17E-14*	1.65E-21*	1.19E-20*	1.04E-15*	1.26E-08*	1.53E-09*	0.000641	2.3E-07*	4.56E-06*	8.44E-11*	6.86E-12*
**rs776746**	CYP3A5	1.44E-20*	1.5E-18*	1.25E-18*	1.82E-21*	5.24E-21*	1.18E-37*	1.94E-33*	3.9E-43*	2.02E-40*	2.85E-40*	8.23E-39*	4.59E-45*	2.21E-13*	9.13E-17*	1.43E-09*	1.67E-14*	4.33E-06*	2.47E-06*	6.55E-07*	8.03E-08*	3.2E-07*	3.69E-22*	3.61E-20*	5.26E-21*	1.44E-19*	1.24E-20*
rs2242480	CYP3A4						2.62E-23*	1.64E-15*	3.89E-36*	1.69E-28*	1.47E-36*	6.57E-33*	1.5E-30*			1.35E-07*		6.01E-14*	3.6E-12*	5.94E-12*	2.02E-09*	1.22E-11*					
rs1805123	KCNH2													0.000824			1.21E-05*	1.31E-06*		1.77E-07*	3.87E-08*	2.29E-09*	2.02E-09*	1.52E-06*	2.17E-08*	0.00023	2.36E-05*
rs4646244	NAT2																							0.00034			3.97E-05
rs4271002	NAT2						0.000159		3.3E-10*	**3.72E-10**	**2.07E-05**	0.000331	4.77E-06*	0.000636				1.74E-06*							0.000333		
rs1041983	NAT2															1.66E-05*		0.000919	0.000817								
rs1801280	NAT2						4.89E-14*	4.38E-14*	2.97E-14*	6.83E-19*	9.29E-22*	4.03E-11*	2.25E-11*	2.47E-21*	8.41E-19*	6.89E-15*	4.17E-22*	2.27E-25*	1.72E-27*	3.07E-27*	1.97E-30*	1.73E-26*	4.01E-19*	1.06E-18*	8.1E-20*	6.31E-25*	3.64E-15*
rs1799929	NAT2						4.69E-11*	5.21E-11*	1.75E-08*	1.49E-15*	8.89E-19*	9.21E-08*	2.85E-06*	4.54E-20*	4.43E-18*	3.32E-14*	2.5E-19*	3.12E-25*	5.32E-26*	9.84E-26*	2.87E-30*	1.73E-26*	6.28E-18*	3.27E-16*	5.98E-18*	9.49E-22*	3.69E-14*
rs1799930	NAT2															0.00019								0.000563			1.56E-05*
rs1208	NAT2						1.18E-20*	2.46E-17*	3.46E-22*	1.32E-27*	1.45E-27*	4.05E-19*	2.65E-22*	6.25E-22*	4.12E-23*	6.89E-15*	5E-22*	6.43E-24*	1.92E-25*	8.03E-26*	1.73E-30*	4.24E-27*	9.85E-22*	1.06E-18*	9.54E-20*	1.22E-25*	2.87E-17*
rs1799931	NAT2				0.000153		2.09E-08*	3.35E-05*	6.27E-11*	8.62E-12*	1.51E-11*	8.44E-07*	4.04E-09*	7.83E-06*			2.98E-05*	3.4E-12*	1.03E-08*	1.72E-09*	1.6E-09*	3.59E-11*		2.94E-07*	9.44E-07*	1.38E-06*	1.99E-05*
rs1495741	NAT2		3.08E-07*		5.51E-08*																5.75E-05			0.000771		6.3E-06*	7.45E-05
rs2115819	ALOX5		0.000363				1.65E-37*	8.62E-25*	4.45E-37*	5.67E-41*	9.79E-33*	1.05E-31*	5.94E-40*	3.77E-16*	9.55E-11*	9.96E-07*	1.12E-14*	5.62E-24*	3.92E-19*	3.07E-18*	6.98E-20*	8.9E-21*	9.03E-16*	6.62E-23*	1.72E-20*	9.48E-16*	2.24E-14*
rs4244285	CYP2C19						8.78E-08*	2.52E-06*	6.75E-05	3.23E-10*	0.00012	6.92E-06*	1.11E-07*	9.77E-11*	2.52E-07*	4.15E-14*	8.47E-10*	2.17E-09*	0.000117	5.20E-08*	7.41E-09*	4.51E-13*					
rs1057910	CYP2C9																							5.21E-07*	0.000111	0.000272	
rs11572103	CYP2C8						1.57E-16*	5.93E-10*	7.08E-15*	8.71E-19*	3.56E-09*	5E-12*	1.60E-14*														
rs7909236	CYP2C8													6.35E-18*	1.175E-16*	1.77E-22*	1.7E-08*	1.45E-16*	3.59E-15*	1.16E-11*	3.98E-10*	2.96E-09*	1.61E-09*	3.5E-14*	3.03E-12*	1.49E-09*	3.17E-06*
rs17110453	CYP2C8						60E-20*	2.07E-14*	1.87E-20*	2.78E-24*	5.66E-22*	1.4E-19*	1.07E-22*	8.29E-11*	2.41E-07*	5.14E-14*	5.74E-10*	1.31E-11*	0.000358	6.93E-10*	0.000121	4.47E-11*					
rs3813867	CYP2E1		**3.59E-06**	0.000102	0.000102	2.78E-06*																					
rs2031920	CYP2E1		8.07E-05			0.0003	5.35E-05		3.99E-05	1.02E-05*	3.99E-05	0.000158	1.66E-05*											0.000331	0.000364	0.000634	0.000111
rs6413432	CYP2E1	4.21E-19*	1.31E-16*	1.45E-15*	3.14E-16*	1.66E-19*				0.000271			0.000531	6.57E-10*	4.59E-08*	9.02E-09*	2.97E-09*	1.63E-06*	**1.26E-05**			9.51E-05	8.46E-12*	1.83E-17*	7.76E-12*	9.13E-08*	2.51E-12*
rs2070676	CYP2E1						3.81E-30*	1.06E-17*	1E-28*	5.52E-30*	2.7E-35*	3.78E-31*	7.09E-28*														
rs5219	KCNJ11		0.000522				4.08E-16*	5.26E-07*	1.86E-24*	2.18E-25*	1.16E-23*	7.22E-22*	4.56E-26*	6.22E-05									0.000384	0.000294		3.67E-07*	
rs1801028	DRD2																							1.31E-13*			9.84E-09*
rs2306283	SLCO1B1				9.67E-06*									8.87E-16*	5.47E-20*	2.85E-16*	5.37E-13*	1.5E-21*	3.18E-19*	3.33E-24*	2.97E-22*	4.46E-25*	3.98E-10*	1.37E-11*	7.78E-09*	1.72E-17*	2.41E-11*
rs4516035	VDR							0.000251						1.88E-18*	9.22E-15*	3.1E-09*	7.2E-25*	2.88E-25*	7.28E-39*	1.17E-28*	3.93E-26*	4.94E-31*	1.74E-10*	3.7E-11*	1.81E-10*	1.72E-15*	7.16E-13*
rs762551	CYP1A2								0.000364		1.00E-05*					1.13E-05*								0.000511			2.42E-05*
rs2472304	CYP1A2				1.73E-05*									2.83E-16*	1.62E-07*		2.04E-24*	2.27E-36*	7.1E-29*	1.44E-34*	2.5E-33*	1.03E-26*	0.000626			2.56E-06*	
rs750155	SULT1A1									0.00056						**3.18E-23**							0.000453				1.96E-07*
rs1800764	ACE				5.34E-05		2.61E-33*	8.82E-23*	1.09E-40*	1.94E-46*	1.05E-35*	2.63E-43*	7.78E-46*	4.25E-05			1.34E-05*	6.39E-06*	4.04E-05	0.000366	0.000415	8.38E-08*		3.48E-05*			
**rs4291**	ACE	3.00E-30*	1.28E-35*	5.55E-31*	2.57E-26*	4.86E-35*	1.33E-28*	1.67E-29*	2.72E-30*	8.3E-26*	4.86E-35*	6.79E-26*	3.69E-35*	3.1E-29*	1.28E-34*	4.75E-42*	9.11E-29*	2.72E-30*	1.42E-24*	1.18E-31*	6.37E-28*	5.39E-31*	3.96E-29*	8.23E-28*	2.65E-35*	6.22E-30*	1.00E-28*
rs4267385	ACE						1.22E-24*	2.4E-15*	3.40E-26*	4.89E-32*	2.33E-33*	3.63E-30*	4.04E-30*	5.61E-06*			3.09E-09*	2.69E-10*	1.64E-09*	1.40E-11*	1.79E-12*	7.04E-21*				0.000955	
rs2108622	CYP4F2								4.34E-05				0.000162								2.04E-06*	2.69E-05*	1.82E-08*	1.99E-10*	2.68E-08*	1.38E-07*	1.96E-09*
**rs3093105**	CYP4F2	1.6E-55*	3.54E-51*	3.51E-54*	1.79E-58*	8.09E-59*	1.14E-36*	1.95E-29*	4.42E-32*	2.15E-40*	4.26E-37*	2.84E-35*	3.52E-34*	2.57E-43*	1.25E-41*	7.05E-55*	1.53E-43*	7.84E-44*	8.51E-49*	1.6E-40*	6.4E-34*	1.29E-38*	4.65E-44*	1.27E-45*	1.55E-41*	1.48E-41*	1.25E-45*
rs8192726	CYP2A6													0.000189			0.000248			0.000374	0.000723						
**rs1051298**	SLC19A1	1.51E-07*	2.66E-09*	0.000482	3.97E-08*	2.45E-06*	7.14E-08*	1.6E-06*	2.36E-06*	3.37E-11*	4.68E-09*	4.73E-07*	4.55E-07*	6.37E-09*	1.09E-15*	8.09E-15	6.04E-09*	6.48E-11*	5.13E-09*	5.5E-14*	4.51E-11*	2.71E-10*	1.94E-06*	7.95E-08*	2.61E-07*	3.27E-07*	2.79E-09*
rs1051296	SLC19A1																										
rs1131596	SLC19A1						6.91E-11*		0.000115	2.6E-16*	1.59E-12*	7.5E-11*	4.12E-10*		2.88E-06*	8.07E-05*				9.78E-06*	0.000809					0.00067	
**rs1065852**	CYP2D6	4.20E-16*	1.82E-13*	1.88E-17*	6.28E-22*	7.67E-12*	4.99E-42*	2.82E-38*	3.62E-50*	2.33E-48*	4.31E-58*	2.22E-39*	1.39E-48*	2.14E-38*	1.76E-37*	8.68E-51*	8.43E-41*	4.66E-30*	3.71E-42*	1.47E-31*	2.16E-39*	2.84E-35*	1.95E-31*	3.3E-41*	1.17E-38*	6.16E-47*	2.88E-42*

Bold indicates*p < 0.05/(52×26) indicates statistical significance

Our research results show that rs776746 (*CYP3A5*), rs4291 (*ACE*), rs3093105 (*CYP4F2*), rs1051298 (*SLC19A1*) and rs1065852 (*CYP2D6*) are the five important VIP variants, and their drug-related information is shown in Table 4. Rs776746 (*CYP3A5*) is mainly related to the dose and metabolism/pharmacokinetics of tacrolimus in the East Asian populations. Rs4291 (*ACE*), which plays a functional and important role in captopril, is related to the toxic effects of aspirin in the East Asian populations and is related to amlodipine,chlorthalidone,and lisinopril in the mixed populations. Rs3093105 (*CYP4F2*) plays a metabolic/pharmacokinetic role in vitamines. In the European populations, rs1051298 (*SLC19A1*) plays an effective and crucial role in the bevacizumab pemetrexed drug and the pemetrexed drug in the mixed populations. In the East Asian populations, rs1065852 (*CYP2D6*) plays a metabolic/pharmacokinetic role in alpha-hydroxymetoprolol and is related to citalopramescitalopram in the European populations. This gene is also closely related to iloperidone. In clinical medication, SNPs at the same variant have different effects on the types and effects of drugs in the different populations, which should be fully and carefully considered.

**Table 4:** Significant VIP variants and drug-related information in the Wa population

Variant	PMID	Molecules	*P*-value	#Of case	#Of control	Study size	Bipgeographical group	Paper discusses	Gene
rs776746	16421475	tacrolimus		53		53	East Asian	metabolism/PK	CYP3A5
rs776746	23073468	tacrolimus	0.016	25		25	East Asian	dosage	CYP3A5
rs776746	21677300	tacrolimus	0.025	209		209	Mixed Population	toxicity	CYP3A5
rs776746	16424824	tacrolimus		201		201	East Asian	metabolism/PK	CYP3A5
rs776746	24120259	tacrolimus	< 0.0001	68		68	East Asian	metabolism/PK	CYP3A5
rs4291	27546928	captopril	0.029	190		190	Unknown	efficacy	ACE
rs4291	18727619	aspirin	0.015	81	231	312	East Asian	toxicity	ACE
rs4291	20577119	amlodipinechlorthalidonelisinopril	0.0014	9309		9309	Mixed Population	other	ACE
rs3093105	20861217	vitamine	< 0.003				Unknown	metabolism/PK	CYP4F2
rs1051298	19841321	bevacizumabpemetrexed	0.01	48		48	European	efficacy	SLC19A1
rs1051298	24732178	pemetrexed	0.016	136		136	Mixed Population	efficacy	SLC19A1
rs1065852	10223777	alpha-hydroxymetoprolol	< 0.05	40		40	East Asian	metabolism/PK	CYP2D6
rs1065852	24528284	citalopramescitalopram	2.00E-16	435		435	European	other	CYP2D6
rs1065852	23277250	iloperidone	0.028	128		128	Unknown	other	CYP2D6

*p* < 0.05 indicates statistical significance

**Fig. 1:**
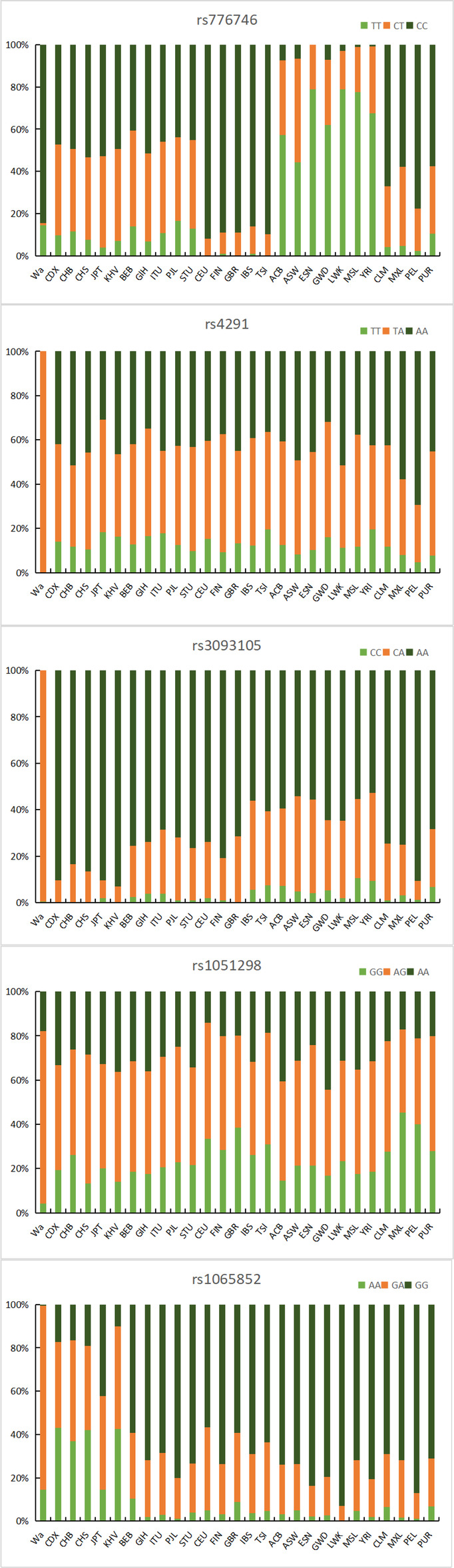
Genotype frequency of significant VIP variants in 27 global populations

**Fig. 2:**
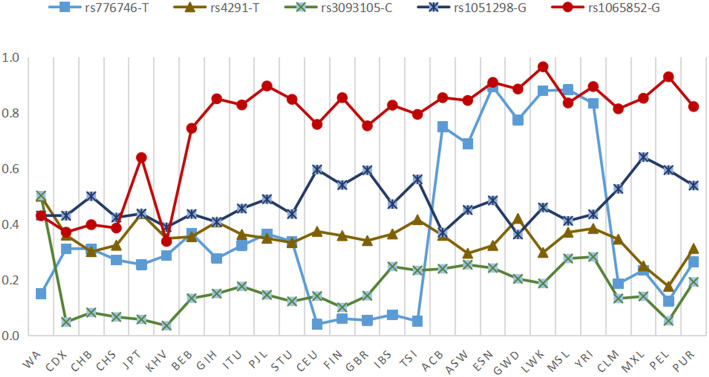
Distribution of alleles with significant VIP variants in 27 global populations

We combined the calculated allele frequencies with previously published data from the global population, and then conducted a comprehensive analysis of the above several loci. Figure 1 shows that the frequency of the GA genotype of rs1065852 is the highest one (85%) in the Wa population; the frequency of the GG genotype of rs1065852 and the CT genotype of rs776746 is the lowest in the Wa population, but the highest is in the African population. In the Wa population, the TA genotype frequency of rs4291 is 1.00%, the CA genotype frequency of rs3093105 is 99.5%, and the AG gene of rs1051298 has a type frequency of 77.9%, which is significantly higher than that of the other populations, showing that the genotype frequencies of the same SNPs in different races are diverse. Figure 2 clearly shows that rs4291-T and rs3093105-C are the highest among the Wa population, with a frequency ranging from 40% to 60%, while rs1065852-G is the lowest among the East Asian population, with a frequency ranging from 34% to 64%. Rs776746-T is the highest in the African population and the lowest in the Wa population; the frequency of rs1051298-G in the East Asian population is 38%-50%, which is lower than that of in the American population. In short, the distribution of alleles is different in each ethnic group, which indicates that there are some differences in genetic background.

## Discussion

Pharmacogenomics refers to gene-based testing to give the appropriate medicine to different patients at the right dose, thereby maximizing the efficacy and minimizing toxicity, thus improving the goal of personalized medicine [11]. In our study, we selected 52 variant genes related to drug response in the Yunnan Wa ethnic group from PharmGKB and compared the results with the other 26 populations distributed worldwide. The research results are not only enriched the knowledge of Wa pharmacogenomics but also laid a certain theoretical foundation for individualised medication. In our study, we found that the frequency of *CYP3A5* rs776746, *ACE* rs4291, *CYP4F2* rs3093105, *SLC19A1* rs1051298, and *CYP2D6* rs1065852 in the Wa population is higher than the other 26 populations from the 1000 Genomes Project. There are significant differences in the genotype frequency and allele distribution of these VIP variants. For the reason of these differences, we should also consider some factors affecting allele frequency distribution, such as genetic mutation, natural selection, genetic drift, and individual migration between populations. Wa people in the Yunnan Province of China may have special living environment and eating habits, as well as an unique geographical location.

*CYP3A5* is located in chromosome 7q21-q22.1, encoding an enzyme of the *CYP3A* subfamily. The most common nonfunctional variant is CYP3A5*3. The status of CYP3A5*3 is determined by the rs776746-derived allele, that is, the change of intron 3 from A to G [12]. Tacrolimus is an immunosuppressant of calcineurin inhibitors which can prevent allograft rejection in solid organ transplant recipients [13, 14]. After studying the effect of *CYP3A5* (rs776746) on the concentration/doses (C/Ds) of tacrolimus and the long-term prognosis of Chinese heart transplantation, Liu et al. [15] found that *CYP3A5* nonexpressors (CYP3A5*3/*3) did not expressed in all point of time. The C/Ds of crolimus are significantly higher than that of expressers (CYP3A5*1/*3), so nonexpressors have higher tacrolimus C/Ds, and expressers tend to have the worse long-term prognoses. In our study, we found that *CYP3A5* rs776746 is more significant in the Wa population compared with the other 26 populations, which is related to tacrolimus dose and metabolism/pharmacokinetics in the East Asian population which indicates that the factor should be fully considered when performing tacrolimus therapy to help to determine the appropriate dose.

Cytochrome P450 4F2 (*CYP4F2*) is an omega-hydroxylase and the only enzyme which is currently showed to metabolize vitamin E in the human body [16]. There are two common genetic variants (V433M, rs2108622 and W12G, rs3093105) that can change its activity. *CYP4F2* gene polymorphisms affects vitamin E to improve the liver of nonalcoholic fatty liver disease children and adults who participated in the Treatment of Nonalcoholic Fatty Liver Disease in Children and Pioglitazone versus Vitamin E versus Placebo for the Treatment of Nondiabetic Patients with Nonalcoholic Steatohepatitis Histology, but there are obvious individual differences in its efficacy [17]. Studies have shown that the W12G mutant has increased enzymatic activity on tocopherols and tocotrienols, while the V433M mutant has reduced enzymatic activity on tocopherols. There is no reduced enzymatic activity on tocotrienols. The influence of these SNPs on vitamin E status and the response of the human body to vitamin E supplementation has an important and obvious clinical significance [16]. The MAF W12G variants in the European and African American populations have been reported to be 11% and 21%, respectively. By using the Asian combined sampling group (Chinese and Japanese HapMap data sets), the W12G variants, the MAF of the body is 6% [18]. The results shows that in the Wa population, the C allele frequency of rs3093105 is 40%-60%, which is higher than that of the other populations in China. Not only that, this gene can affect the metabolism/pharmacokinetics of vitamin E. Therefore, the fact that patients supplemented vitamin E and clinicians had fully understanding its status will help clinicians to better individualize treatment.

The canonical RefSeq *CYP2D6* gene spans approximately 4,400 nucleotides, including 9 exons, and is encoded on the negative strand of the chromosome 22q13.2 [19]. *CYP2D6* polymorphisms can affect the metabolism of alpha-hydroxymetoprolol [20], citalopramescitalopram [21], and iloperidone [22]. Drug dosage can be recommended according to the metabolism of *CYP2D6*. A previous study of atorvastatin in the treatment of ischemic stroke found that the G allele of rs1065852 (*CYP2D6*) had a better lipid-lowering effect, and patiebts carrying the GG genotype had a better effect on atorvastatin treatment reaction. For example, patients with insulin resistance who carry the GG genotype should be considered to reduce atorvastatin use to avoid the drug reactions [23]. Li et al. [24] reported that in the Han population with lung cancer in Northwestern China,the most significant correlation is the A allele of *CYP2D6* rs1065852 and the AA genotype, which can increase the cancer risk. Sun et al. [25] showed that the G allele in the *CYP2D6* rs1065852 may be related to the efficacy of labetalol in the treatment of early-onset preeclampsia. This study found that the G allele frequency of rs1065852 in the East Asian population was 34%-64%, and the frequency of the GG genotype in the Wa population was 0.5%, which were much lower than the other populations. Therefore, when clinicians use drugs to treat related diseases, the optimal dose of the drug should be based on the specific genotype of the individual patient to maximize the therapeutic effect.

Angiotensin-converting enzyme (*ACE*), encoded by the *ACE* gene, is located in 17q23, consists of 28 exons and 25 introns. *ACE* participates in the renin-angiotensin-aldosterone system (RAAS), which affects salt retention a protein for water balance and blood vessels; therefore, RAAS controls blood pressure, and drugs that inhibit this enzyme are effective in treating high blood pressure [26]. Migdalov et al. [27] demonstrated that captopril can be used to lower blood pressure by inhibiting *ACE*. Studies have shown that through the changes in fasting urea and creatinine over one year of dementia caused by Alzheimer’s disease (AD), the use of angiotensin converting enzyme inhibitors has found to be effective for carriers of rs1800764 CT/rs4291 AA. Though having a protective effect, changes in creatinine is harmful to carriers of rs1800764 CT/rs4291 AT [28]. Our study found that the TA genotype frequency was 1.00 in the Wa population, which was higher than that of in the other populations, while the AA genotype frequency was the lowest, which indicated that the optimal dose of *ACE* inhibitor should be based on the specific genotype of the individual Wa patients.

The *SLC19A1* gene encodes a folate transporter and is involved in the regulation of intracellular folate concentration [29]. Studies have shown that folate carrier protein 1 (*SLC19A1*) affects the transport process of pemetrexed in the body. An analysis of the Han patients with non-small cell lung cancer who were only received pemetrexed treatment showed that the *SLC19A1* rs1051298 (c.*746 C > T) increases the risk of all adverse drug reactions of pemetrexed treatment in different cycles. As with the risk of all adverse reactions, this effect is particularly important in liver injury [30]. Corrigan et al. [31] found that the SNP rs1051298 in the *SLC19A1* gene can affect the overall survival and progression-free survival of patients with advanced non-small cell lung cancer receiving pemetrexed combined with platinum therapy. The results show that compared with the other 26 populations, the Wa population *SLC19A1* rs1051298 is more significant and based on its polymorphism affecting the efficacy of pemetrexed, we can maximize the therapeutic effect of pemetrexed on the Wa patients.

## Conclusions

This study analyzed the differences in genotype frequency and allele distribution between the Wa ethnic group and the other 26 ethnic groups worldwide. Rs776746 (*CYP3A5*), rs4291 (*ACE*), rs3093105 (*CYP4F2*), rs1051298 (*SLC19A1*) and rs1065852 (*CYP2D6*) in the Yunnan Wa population have a higher frequency, which provides a theoretical basis for safe medication and efficacy improvement. Our study complement the pharmacogenomics information of Wa population from Yunnan province and provide valuable information for future studies and better individualized treatments. This study has certain limitations. Due to the small sample size and the unadvanced genotyping technology, it is not able to fully and totally detect less common variants (in fact, variants with potentially important pharmacogenomic markers) that may (erroneously) give negative results, so participants may carry other important DNA variants not detected by the Agene MassARRAY platform. A large number of sample studies are also needed to verify the accuracy of our research.

## Methods

### Study participants

We randomly recruited 200 unrelated Wa adults from the Yunnan province of China. The selected subjects were judged to be in good health according to their medical history and had only Wa ethnic origins in at least the last three generations. In addition, this study was conducted in accordance with the Declaration of Helsinki, and the protocol was approved by the Clinical Research Ethics of Xizang Minzu University. Each participant also signed an informed consent form.

### Variant selection and genotyping

We searched the PharmGKB database and 52 random VIP variants of 27 genes were ultimately selected for our study according to available data on frequency, functionality, and linkage based on published research. The method of operation used was to extract the genomic DNA of peripheral blood according to the GoldMag-Mini whole blood genome DNA Purification Kit (GoldMag Ltd. Xi'an, China). The DNA concentration was measured by a NanoDrop 2000C spectrophotometer (USA). Agena MassARRAY Assay Design 4.0 software (San Diego, California, USA) was used to design multiple SNP MassEXTEND arrays (Gabriel et al., 2008) and to design primers and single base extension primers for the selected sites. The PCR primers for the selected variants are presented in Supplementary Table 1. Following the instructions provided by the manufacturer, we used Agena MassARRAY RS1000 (San Diego, California, USA) to determine the genotype of the SNP. A brief overview of the Agena MassARRAY RS1000 (San Diego, California, USA) method for genotyping were as follows: (1) PCR amplification, (2) SAP purification, (3) iPLEX single base extension reaction, (4) resin exchange, and (5) mass spectrometry detection. Finally, Agena Typer 4.0 software was used for data statistics and analyses (Thomas et al., 2007) [32].

### 1000 Genomes Project

The individual genotype data of the 26 populations were downloaded from the website of the 1000 Genomes Project (http://www.1000genomes.org/) [33]. These 26 populations were: (1) African Caribbean in Barbados (ACB); (2) African Ancestry in Southwest US (ASW); (3) Esan in Nigeria (ESN); (4) Gambian in Western Divisions, The Gambia – Madinka (GWD); (5) Luhya in Webuye, Kenya (LWK); (6) Mende in Sierra Leone (MSL); (7) Colombian in Medellin, Colombia (CLM); (8) Mexican Ancestry in Los Angeles, California (MXL); (9) Peruvian in Lima, Peru (PEL); (10) Puerto Rican in Puerto Rico (PUR); (11) Chinese Dai in Xishuangbanna, China (CDX); (12) Yoruba in Ibadan, Nigeria (YRI); (13) Han Chinese South (CHS); (14) Japanese in Tokyo, Japan (JPT); (15) Kinh in Ho Chi Minh City, Vietnam (KHV); (16) Utah residents with Northern and Western European ancestry (CEU); (17) Finnish in Finland (FIN); (18) British in England and Scotland (GBR); (19) Iberian populations in Spain (IBS); (20) Toscani in Italy (TSI); (21) Bengali in Bangladesh (BEB); (22) Gujarati Indians in Houston, Texas (GIH); (23) Indian Telugu in the UK (ITU); (24) Punjabi in Lahore, Pakistan (PJL); (25) Sri Lankan Tamil in the UK (STU), and (26) Han Chinese in Beijing, China (CHB).

### Statistical analyses

Microsoft Excel and SPSS 20.0 statistical software packages were used to perform Hardy-Weinberg equilibrium (HWE) analysis and χ^2^ tests (SPSS, Chicago, IL, USA). The χ^2^ tests were used to evaluate the frequency of variation from HWE in the Wa population for verification. In this study, All *p*-values were two-sided and *p*-values less than 0.05 were considered statistically significant. Next, the Bonferroni multiple adjustment method was used for correction, and *p* < 0.05/(52×26) has a significant difference. Subsequently, we obtained SNPs allele frequencies from the Ensemble database (https://asia.ensembl.org/index.html). Finally, the overall genetic variation pattern of specific loci was analyzed [34].

## Supplementary Information

**Additional file 1: Table S1.** Primer sequence.

## Data Availability

The datasets generated and analyzed during the current study are available in the [figshare] repository, [10.6084/m9.figshare.14782323.v1] and the accession numbers is tianbojin63@163.com.

## Abbreviations

VIP: Very important pharmacogene
SNPs: Single nucleotide polymorphisms
ADR: Adverse drug reaction
CPIC: Clinical pharmacogenetics Implementation Consortium
MAF: Minor allele frequency
C/Ds: Concentration/dose
*ACE*: Angiotensin-converting enzyme
AD: Alzheimer’s disease
ADW: Average daily warfarin
ACE: Angiotensin-converting enzyme
RAAS: Renin-angiotensin-aldosterone system
ACB: African Caribbean in Barbados
ASW: African Ancestry in Southwest US
ESN: Esan in Nigeria
GWD: Gambian in Western Divisions, The Gambia – Madinka
LWK: Luhya in Webuye, Kenya
MSL: Mende in Sierra Leone
CLM: Colombian in Medellin, Colombia
MXL: Mexican Ancestry in Los Angeles, California
PEL: Peruvian in Lima, Peru
PUR: Puerto Rican in Puerto Rico
CDX: Chinese Dai in Xishuangbanna, China
YRI: Yoruba in Ibadan, Nigeria
CHS: Han Chinese South
JPT: Japanese in Tokyo, Japan
KHV: Kinh in Ho Chi Minh City, Vietnam
CEU: Utah residents with Northern and Western European ancestry
FIN: Finnish in Finland
GBR: British in England and Scotland
IBS: Iberian populations in Spain
TSI: Toscani in Italy
BEB: Bengali in Bangladesh
GIH: Gujarati Indians in Houston, Texas
ITU: Indian Telugu in the UK
PJL: Punjabi in Lahore, Pakistan
STU: Sri Lankan Tamil in the UK
CHB: Han Chinese in Beijing, China
HWE: Hardy-Weinberg equilibrium
