# Supplementary material for: Genetic analysis of pharmacogenomic VIP variants in the Wa population from Yunnan Province of China
Source: BMC Genom Data. 2021 Nov 19;22:51. doi: 10.1186/s12863-021-00999-8 (PMC8605568; doi:10.1186/s12863-021-00999-8)
Supplement: Supplementary file 1 — Additional file 1: Table S1. Primer sequence. [file 12863_2021_999_MOESM1_ESM.docx]

**Genetic analysis of pharmacogenomic VIP variants in the Wa population from Yunnan Province of China**

Dandan Li, Linna Peng, Shishi Xing, Chunjuan He, Tianbo Jin

**Supplementary** Table 1 Primer sequence

| SNP ID | 2nd-PCRP | 1st-PCRP | UEP_SEQ |
| --- | --- | --- | --- |
| rs11572325 | ACGTTGGATGTTGTTGTGCCCAAAGTCAGG | ACGTTGGATGCATTACTTGTTCTGATGCTC | GTCAGGAAATATCAGCTTGA |
| rs10889160 | ACGTTGGATGGGATAATGAGAGGAAGTTGC | ACGTTGGATGGTAGATTACATCAGATTCC | GGGAGAAAATCGAACTTTGTG |
| rs1760217 | ACGTTGGATGAAAACCTATTCCCCTGTCTG | ACGTTGGATGCATCTCTCTTGAAACCTGAC | gGTCTGAAATTTAATGCTACACAATG |
| rs1801159 | ACGTTGGATGTGCGCTAGCAAGACCAAAAG | ACGTTGGATGCTCCTATTGATCTGGTGGAC | GCAAGACCAAAAGGATTTA |
| rs1801265 | ACGTTGGATGATCCTGGCTTTAAATCCTCG | ACGTTGGATGCTTGTCTAATTTCTTGGCCG | CACAAACTCATGCAACTCTG |
| rs5275 | ACGTTGGATGCACTGTCGATGTTTCCAATG | ACGTTGGATGTGCACTGATACCTGTTTTTG | agGTTTGAAATTTTAAAGTACTTTTGGT |
| rs20417 | ACGTTGGATGACAGGGTAACTGCTTAGGAC | ACGTTGGATGACTGTTCTCCGTACCTTCAC | ggattAGGAGAATTTACCTTTCCC |
| rs12139527 | ACGTTGGATGGTTGCCATGATGAAGTTTGC | ACGTTGGATGAAGACCCCACTCAGCCTACT | GTTTGCATCAGCTGCC |
| rs3850625 | ACGTTGGATGGGAAGTTCTACGCCACATTC | ACGTTGGATGTGGGCCGATAGCCATAATAC | TCCGGAAGTTCATGAAA |
| rs2306238 | ACGTTGGATGGGGAGTACTGGATAGAGTC | ACGTTGGATGAGATGGGTCTAATCCCTCAG | tttTAGAGTCCTCTAGTCTAGGT |
| rs2231142 | ACGTTGGATGTGATGTTGTGATGGGCACTC | ACGTTGGATGGTCATAGTTGTTGCAAGCCG | aTGACGGTGAGAGAAAACTTA |
| rs2231137 | ACGTTGGATGTCAGGTCATTGGAAGCTGTC | ACGTTGGATGGATGTCTTCCAGTAATGTCG | gcatgGGTGTTTCCTTGTGACA |
| rs698 | ACGTTGGATGAGAGCGAAGCAGGTCAAATC | ACGTTGGATGTCCCCAAACTTGTGGCTGAC | CTTCATTTATTTTTTCAAAAGGTAAAA |
| rs776746 | ACGTTGGATGGTAATGTGGTCCAAACAGGG | ACGTTGGATGATGTACCACCCAGCTTAACG | cgCCAAACAGGGAAGAGATA |
| rs2242480 | ACGTTGGATGTGCTAAGGTTTCACCTCCTC | ACGTTGGATGGCAGGAGGAAATTGATGCAG | ttaACCTCCTCCCTCCTTCTCCATGTA |
| rs1805123 | ACGTTGGATGGCAAGCGCAAGTTGTCCTTC | ACGTTGGATGTAAAGCAGACACGGCCCAC | agcaGCAGGCGCACGGACA |
| rs4646244 | ACGTTGGATGTACATACCTCTGGCATGCTG | ACGTTGGATGTTGTAGTCCATCTGCCCAAG | GCATGCTGCCACATGA |
| rs4271002 | ACGTTGGATGCCCAAAGGTAACACACAATG | ACGTTGGATGGCAGAAACAAAGCCATATGA | aATGCATGTGGTATAAGTGT |
| rs1041983 | ACGTTGGATGAGACCACAATGTTAGGAGGG | ACGTTGGATGCAGGAGAAGGTGAACCATGC | CAATGTTAGGAGGGTATTTTTA |
| rs1801280 | ACGTTGGATGCAAATACAGCACTGGCATGG | ACGTTGGATGGACCCAGCATCGACAATGTA | TCCTGCAGGTGACCA |
| rs1799929 | ACGTTGGATGGGCAGGAGATGAGAATTAAG | ACGTTGGATGTTCTGCTTGACAGAAGAGAG | cttcgCTCTCCTGATTTGGTCCA |
| rs1799930 | ACGTTGGATGCCTGCCAAAGAAGAAACACC | ACGTTGGATGAAGATGTTGGAGACGTCTGC | ccagACTTATTTACGCTTGAACCTC |
| rs1208 | ACGTTGGATGACAATACAGATCTGGTCGAG | ACGTTGGATGATTTCTCCCCAAGGAAATC | GGTTGAAGAAGTGCTGA |
| rs1799931 | ACGTTGGATGGGGTGATACATACACAAGGG | ACGTTGGATGGGAAGAGGTTGAAGAAGTGC | CCTTATTCTAAATAGTAAGGGAT |
| rs1495741 | ACGTTGGATGTCTCTCAGGAAAGGAGCAAA | ACGTTGGATGGGCCTCACATGGTCACTTC | tCTGAAGGATGATTTTCATAATAAT |
| rs2115819 | ACGTTGGATGTTTGTGTAACACTGGGATGG | ACGTTGGATGGCCACAGGAGATTTTAGTTC | ccaTGGGATGGAAAGGGT |
| rs4244285 | ACGTTGGATGGCAATAATTTTCCCACTATC | ACGTTGGATGTCCATCGATTCTTGGTGTTC | CCCACTATCATTGATTATTTCCC |
| rs1057910 | ACGTTGGATGATGCAAGACAGGAGCCACAT | ACGTTGGATGTGTCACAGGTCACTGCATGG | ccctACGAGGTCCAGAGATAC |
| rs11572103 | ACGTTGGATGGCTAATATCTTACCTGCTCC | ACGTTGGATGTGGATGTTAACAATCCTCGG | TTACCTGCTCCATTTTGA |
| rs7909236 | ACGTTGGATGTTTCTCCATCATCACAGCAC | ACGTTGGATGGCCAATCTAGGAGATTCTGG | cccTCACAGCACATTGGAA |
| rs17110453 | ACGTTGGATGACACTGATTTCCCTCAAGGT | ACGTTGGATGCTGTGATGATGGAGAAACAC | ccTTCCCTCAAGGTCATAAA |
| rs3813867 | ACGTTGGATGCAACGCCCCTTCTTGGTTCA | ACGTTGGATGGCAAGTCATTGGTTGTGCTG | CCTTCTTGGTTCAGGAGAG |
| rs2031920 | ACGTTGGATGGTTCTTAATTCATAGGTTGC | ACGTTGGATGCAAGTGATTTGGCTGGATTG | TTAATTCATAGGTTGCAATTTT |
| rs6413432 | ACGTTGGATGTCCCAAGTAACTGGGCCACA | ACGTTGGATGCTGTGCCCAGCCAAAATAAT | aCACACCCAGCTGATTAAAAATT |
| rs2070676 | ACGTTGGATGATCCTTCACTAAGCAACTCC | ACGTTGGATGGAAACCCCCAGTGAAGAATG | aatgCACTAAGCAACTCCTTCAACT |
| rs5219 | ACGTTGGATGCGTTGCAGTTGCCTTTCTTG | ACGTTGGATGAGGAATACGTGCTGACACGC | cggtCACGGTACCTGGGCT |
| rs1801028 | ACGTTGGATGAGCCACCACCAGCTGACTCT | ACGTTGGATGATTCTTCTCTGGTTTGGCGG | cacccGCTGACTCTCCCCGACCCGT |
| rs2306283 | ACGTTGGATGACCTTTTCCCACTATCTCAG | ACGTTGGATGGATGTTCTTACAGTTACAGG | ttGATGTTGAATTTTCTGATGAAT |
| rs4516035 | ACGTTGGATGTTTCCTCCTCTGTAAGAGGC | ACGTTGGATGCAGTCGATGACCTCCTTTAG | cggcAGAGGCGAATAGCAAT |
| rs762551 | ACGTTGGATGCTAAGCTCCATCTACCATGC | ACGTTGGATGGAATCTTGAGGCTCCTTTCC | CTACCATGCGTCCTG |
| rs2472304 | ACGTTGGATGAACCCTATAGCCAGGAGAAG | ACGTTGGATGACACAGCAGGCACATAACAG | CCAGGAGAAGCCTTGA |
| rs750155 | ACGTTGGATGCATCTCCACTGGGTGGAGG | ACGTTGGATGAGAGTCCAGCTGCACTGAG | GGGTCCCAGCAGGAA |
| rs1800764 | ACGTTGGATGAAGACAACCCATGGGATGAG | ACGTTGGATGCTTGGAATGTACCCACTGAG | TGCAAAGTATGTACAGCA |
| rs4291 | ACGTTGGATGCAGAGGAAGCTGGAGAAAGG | ACGTTGGATGTCGGGTGTTCCGGCAAACTG | ccccGGGCCTCCTCTCTTT |
| rs4267385 | ACGTTGGATGAGCCAGGTTATCTCTCTAGG | ACGTTGGATGGATTGCACAGCCCATAAGAG | tcttaTAACTTGACCTCTTATGTTCAC |
| rs2108622 | ACGTTGGATGCTAGGAGCCTTGGAATGGAC | ACGTTGGATGTGCCTCATCAGTGTTTTCGG | CCTCAGGGTCCGGCCACA |
| rs3093105 | ACGTTGGATGATGTCCCAGCTGAGCCTGT | ACGTTGGATGTGTCATAGAAGGCGTAGGTC | agaCTGTCCTGGCTGGGCCTC |
| rs8192726 | ACGTTGGATGTCCTCGTCCTGGGTGTTTTC | ACGTTGGATGGGCTTCCTCATCGACGCCC | TCCTTCTCCTGCCCC |
| rs1051298 | ACGTTGGATGTGGCACATACCAAGGCCAG | ACGTTGGATGCTTCTGAAGTGTGTCCATCC | gAAGGCCAGCACGTCC |
| rs1051296 | ACGTTGGATGTCTGAAGTGTGTCCATCCTG | ACGTTGGATGATACCAAGGCCAGCACGTC | tAGCTGCTCCCACACT |
| rs1131596 | ACGTTGGATGACCATCCTGCTCAGGCCAC | ACGTTGGATGATCTTCCAAGGTGCCCTGAC | GGGGACGAAGGTGAC |
| rs1065852 | ACGTTGGATGTGCTCCTGGTGGACCTGATG | ACGTTGGATGTGGAAGTCCACATGCAGCAG | cccctCTGGGCTGCACGCTAC |
